# The More Extended Use of Muriate of Cocaine

**Published:** 1885-08-20

**Authors:** A. F. Sampson

**Affiliations:** Galveston, Texas


### THE MORE EXTENDED USE OF MURIATE OF COCAINE.

BY A. F. SAMPSON, M.D., GALVESTON, TEXAS.

**Read before the Galveston Medical Club, and published by resolution of the Club.**

The anæsthetic properties of cocaine have so dazzled the minds of the profession that they seem slow to avail themselves of the more extensive therapeutical advantages to be gained from this alkaloid. Cocaine, possessing antiphlogistic properties to a marked degree, by its dual action, viz.: Subduing nerve irritation and capillary engorgement, its range of application to disease must necessarily be great.

Take the vast degrees of catarrhal inflammation that have almost defied treatment heretofore, what are the pathological conditions we have to contend with? A chronic pharyngitis, for example. We find from the chronic engorged capillaries exuding their plasma excessively, that there is an infiltration of the mucous membrane, with its cellular elements distended and swollen, there results a rapid exfoliation of its superficial layer of cells, leaving a denuded, sensitive surface.

That cocaine, locally applied in its physical effects, will meet the therapeutical indications in such conditions, seems evident. I believe a flattering success has been attained by those who have used this drug in a proper manner, in the treatment of catarrhal inflammation.

I will give a brief report of one case that may serve as a test of the efficacy of cocaine in the treatment of chronic catarrhal inflammation.

Mrs. A., aged 50, chronic catarrhal cystitis of twenty years standing. Her life was a burden by the coconmitant symptoms of her disease. Her frequent micturition and constant irritation had deprived her of the interchange of social visits. She had grown despondent after undergoing so many years of treatment without deriving any degree of permanent benefit; her condition had resisted topical applications in the bladder at the hands of a very skilled physician. Emptying the bladder of all urine, and washing it out, I then applied by injection into this viscus a 2 per cent. sol. of mur. cocaine. I soon saw the anæesthetic effects had been gained. Next day found my patient had derived marked relief with considerable dimunition in the amount of mucous in the urine; repeated the application. On visiting my patient the following day found her grateful for the improvement gained. The third day found the bladder evincing but little sensitiveness to the wash. She reported having passed urine but four times in the past twenty-four hours, saying it was from a natural, and not that old burning desire. During the following week I allowed a day to intervene in making my visits and applications. The patient not experiencing the first relapse, her improvement advanced to such a degree of satisfaction that she claimed herself cured. The urine passed during the night or on rising in the morning, would be standing several hours ere I saw it, though at the end of the second week's treatment it showed no mucous deposit. I gradually lengthened the intervals to see her, until now my visits are spaced five days, with instructions to be immediately notified as soon as any uneasiness may be felt in the bladder. Throughout the entire treatment of this trying case there has not been a single relapse. Though I may not dignify this as a case of a very intractable cystitis, treated with the greatest degree of comfort to the patient and satisfaction to the physician, I am at least warranted to extend this new procedure to your consideration.

In the treatment of *urethritis*, I think this drug, when properly used, will do away with "Ricord's Hell" for physicians. As three-fourths of the *urethral strictures* are due to a localized chronic urethritis, we may see a vast reduction in the number of these troubles by the judicious application of the physiological properties of cocaine.

*The rectum and anus* will naturally come in for their share of benefits. A rectal ulcer by the laws of repair should yield kindly to the effects of cocaine. The teasing and anal fissure will evidently command the use of this alkaloid.

In acute laryngitis or the majority of laryngeal troubles, we have in the physiological action of a cocaine spray the scientific treatment. Why not extend its effects to the trachia?

In tonsilitis, the rapidity with which the engorged painful gland is relieved, shows the beautiful action of cocaine. Why not its effects to kindred structures?

In the various forms of pharyngitis cocaine should not be denied its advantages.

The inflammatory affections of the nasal surfaces, let us leave to the care of our specialist, except in hay fever and more extensively in acute coryza. (Vide Med. Record, No. 20, Vol. 26.)

In gastritis, from the close pathological relations that exists between the mucous membrane of the stomach and the œsophagus, though I may appear to be advancing into somewhat obscure quarters, still I would not hesitate using a properly designed capsule of cocaine in trying to secure its desirable local physiological action in gastritis.

In gynæcology, I expect to see quite a demand for cocaine, besides its benefits in operative procedure, for its antiphlogistic value, its therapeutical demands will necessarily be great. The operation for curetting should be shortly anticipated by and injection of mur. cocaine on the endometrium, from which there would result no pain and less hemorrhage. In the treatment of endo-cervical catarrh, cocaine will play an important part. Deep hypodermic injections should be borne in mind in the treatment of ovarian neuralgia.

In endo-metritis fungosa, most generally found in cases of subinvolution, this altered endometrium is the result of chronic congestion of the uterus. Here the effects of cocaine are as plainly indicated as the simplicity of its application. To the cervix in the first stage of labor it is said to save a paturient patient a great deal of suffering. Why not continue its happy effects to the vaginal walls and the vulva? thus having, as seems evident, merited the full gratitude of our tried patients.

For fissures and excoriations of the nipples will cocaine be welcomed with true delight.

In teething of infants, I advised the use of cocaine in this vexed problem in December, 1884, though no opportunity presenting itself for me to directly watch its effects; however, I was gratified to see in the Medical Record of February 14, 1885, a report of its use by Dr. Baker on his own child with the happiest results.

This opens a wide field for the benefits to be derived from cocaine. It gives me pleasure to contemplate thus so simply controlling the diarrhœa and other reflex troubles from teething.

In neuralgias I expect to see the hypodermic use of cocaine supercede morphia. In pertussis, I anticipate the efficiency of a cocaine spray.

Would that some genius could carry the local physiological effects of cocaine to Peyers-patches, then would my enthusiasm lead me to regard the drug favorably in typhoid fever.

To the dermatologists the powers of cocaine cannot go uninvited. To the opthalmologists, who have championed Koller's boon, in having seized upon its aid with such avidity, I do not think I can advance a suggestion.

The influence that cocaine will exercise upon new growth is a field unexplored. When we consider that new capillaries are developed in nearly all new growths; that the production of most new growths depended upon a somewhat persistent local irritation, giving rise to perverted nutrition of the parts; that new growths depends upon the formation of new capillaries for their maintenance, these increasing in numbers and size proportionately with the advancement of the growth; that by constricting the calibre of the capillaries we can obliterate them (a most marked example of this was shown in Dr. J. F. Y. Paine's paper, read before the State and National Medical Association, where a tremendious subserious fibroid tumor was almost obliterated under the starving influence of ergot); that in deriving the etiology of epithelioma some such cause as above stated has been found invariably productive of this growth. We see that we have two important elements to combat in our therapeutical efforts, viz.: Nerve irritation and increased blood supply to the parts. The local physiological action of cocaine logically suggests itself. Thus theorizing, that in the physiological action of cocaine, we have an agent that exerts a controlling influence over the essential conditions in establishing and maintaining the development of new growths, I was prompted to offer to Dr. Geo. F. Shrady, editor of the New York Medical Record, an embodiment of these views as a physiological explanation to reconcile Gen. Grant's present marked improved condition with the gloomy announcements that had been bulletined by his physicians, thus bringing down the severe, lampooning criticisms of the laity upon the heads of the medical profession. This is the only published case of epithelioma upon which cocaine has been in any degree used, it being used solely for its anæsthetic effects, and we can but infer that its use was very irregular ; however, we have seen a check in the advancement of the cancer that gives some coloring of importance to my theory. While my enthusiasm for cocaine does not carry me to a degree of vaunting it as a *cancer* cure, I am at least warranted in suggesting it as a scientific therapeutical agent in the treatment of the *bete-noir* to our profession.

By following up the above train of suggestions, with the support of the hypodermic needle, the spray, the pencil, and I may add capsules for the stomach, we can comprehend, in a degree, what an extensive and inviting field the therapeutical application of cocaine to diseases opens up to our profession.

In the constitutional use of the drug, I would forego any suggestions, except to call your attention to the indications for large doses in delirium tremens. As a hypnotic, requiring a 5 gr. dose, is rather severe treatment at the present price of the drug—we will have to defer.

The influence that local use of cocaine exhibits in the healing of wounds. I shall endeavor to show how, by the physiological effect of cocaine, wounds are induced to heal by first intention. We are well aware that in an incision, the traumatic irritation, if of any magnitude, calls into existence capillary engorgement with its concomitant transudation. Allow the irritation to remain and the engorgement to advance, then we have the exudation, causing a swelling, that by its pressure exaggerating the pain, the exudation strangling the vitality of the capillaries, we are thus denied the condition of gaining healing by first intention. What must be the desideratum in gaining healing by first intention ? Why overcoming the advanced degrees of inflammation. I can only ask you to grant me what I have demonstrated in my premise as the physiological action of cocaine, and look to the results that have been gained, then I feel that sufficient convincing argument has been made.

In ascertaining the results of wounds healing under the influence of cocaine from my *confreres*, their reports almost universally sum up, healing by first intention, and that in some cases where it could not be expected. What a boon Koller has bestowed upon the surgeon in this effect alone of cocaine is a picture too grand in its magnitude for me to depict. To be able to get healing without a cicatrix, thus erasing the disfiguring lines left by the keen edge of prompt relief, serves to emulate cocaine to a plane equal to Bulwer's most vivid imaginary Elixir of Life.

I think the healing influence of this agent will hold such sway that the surgeon who would operate on a patient under the influence of a general anæsthetic would be wanting in his duty to his patient in disregarding this advantage of cocaine. In burns, especially, where the epidermis is destroyed, the application of cocaine is the most grateful drug indicated.

In forming your impression of cocaine, beware of an inferior article of the drug, or a solution that has been on hand any length of time.—*Courier-Record of Medicine.*

